# MuscleMap: An Open-Source, Community-Supported Consortium for Whole-Body Quantitative MRI of Muscle

**DOI:** 10.3390/jimaging10110262

**Published:** 2024-10-22

**Authors:** Marnee J. McKay, Kenneth A. Weber, Evert O. Wesselink, Zachary A. Smith, Rebecca Abbott, David B. Anderson, Claire E. Ashton-James, John Atyeo, Aaron J. Beach, Joshua Burns, Stephen Clarke, Natalie J. Collins, Michel W. Coppieters, Jon Cornwall, Rebecca J. Crawford, Enrico De Martino, Adam G. Dunn, Jillian P. Eyles, Henry J. Feng, Maryse Fortin, Melinda M. Franettovich Smith, Graham Galloway, Ziba Gandomkar, Sarah Glastras, Luke A. Henderson, Julie A. Hides, Claire E. Hiller, Sarah N. Hilmer, Mark A. Hoggarth, Brian Kim, Navneet Lal, Laura LaPorta, John S. Magnussen, Sarah Maloney, Lyn March, Andrea G. Nackley, Shaun P. O’Leary, Anneli Peolsson, Zuzana Perraton, Annelies L. Pool-Goudzwaard, Margaret Schnitzler, Amee L. Seitz, Adam I. Semciw, Philip W. Sheard, Andrew C. Smith, Suzanne J. Snodgrass, Justin Sullivan, Vienna Tran, Stephanie Valentin, David M. Walton, Laurelie R. Wishart, James M. Elliott

**Affiliations:** 1 Faculty of Medicine and Health, The University of Sydney, Sydney, NSW 2006, Australia; david.anderson1@sydney.edu.au (D.B.A.); claire.ashton-james@sydney.edu.au (C.E.A.-J.); john.atyeo@sydney.edu.au (J.A.); stephen.clarke@sydney.edu.au (S.C.); adam.dunn@sydney.edu.au (A.G.D.); jillian.eyles@sydney.edu.au (J.P.E.); hfen8792@uni.sydney.edu.au (H.J.F.); ziba.gandomkar@sydney.edu.au (Z.G.); sarah.glastras@sydney.edu.au (S.G.); luke.henderson@sydney.edu.au (L.A.H.); claire.hiller@sydney.edu.au (C.E.H.); sarah.hilmer@sydney.edu.au (S.N.H.); brian.kim@sydney.edu.au (B.K.); sarahmaloney18@gmail.com (S.M.); lyn.march@sydney.edu.au (L.M.); margaret.schnitzler@sydney.edu.au (M.S.); justin.sullivan@sydney.edu.au (J.S.); james.elliott@sydney.edu.au (J.M.E.); 2 Division of Pain Medicine, Stanford University School of Medicine, Stanford University, Stanford, CA 94304, USA; kenweber@stanford.edu (K.A.W.II); e.o.wesselink@vu.nl (E.O.W.); 3 Faculty of Behavioural and Movement Sciences, Amsterdam Movement Sciences—Program Musculoskeletal Health, Vrije Universiteit Amsterdam, 1081 BT Amsterdam, The Netherlands; a.l.goudzwaard@vu.nl; 4 Department of Rehabilitation Medicine, University of Oklahoma, Norman, OK 73019, USA; zachary-a-smith@ouhsc.edu; 5 Department of Rehabilitation Medicine, University of Minnesota, Minneapolis, MN 55455, USA; rebecca.abbott@cuanschutz.edu; 6 Faculty of Medicine, Health and Human Sciences, Macquarie University, Macquarie Park, NSW 2109, Australia; aaron.beach@mq.edu.au (A.J.B.); john.magnussen@mq.edu.au (J.S.M.); 7 Disability Prevention Program, Department of Epidemiology and Cancer Control, St. Jude Children’s Research Hospital, Memphis, TN 38105, USA; 8 School of Health and Rehabilitation Sciences, University of Queensland, Brisbane, 4072 QLD, Australia; n.collins1@uq.edu.au (N.J.C.); melinda.smith@uq.edu.au (M.M.F.S.); s.oleary@uq.edu.au (S.P.O.); laurelie.wishart@health.qld.gov.au (L.R.W.); 9 School of Health Sciences and Social Work, Griffith University, Brisbane, QLD 4111, Australia; m.coppieters@griffith.edu.au (M.W.C.); j.hides@griffith.edu.au (J.A.H.); 10 Otago Medical School, University of Otago, Dunedin 9016, New Zealand; jon.cornwall@otago.ac.nz (J.C.); phil.sheard@otago.ac.nz (P.W.S.); 11 Faculty of Health Sciences, Curtin University, Perth, WA 6845, Australia; crawford.rj.ac@gmail.com; 12 Department of Health Science and Technology, Aalborg University, Gistrup, 9260 North Jutland, Denmark; edm@hst.aau.dk; 13 Northern Sydney Local Health District, The Kolling Institute, St Leonards, NSW 2065, Australia; 14 Department of Health, Kinesiology & Applied Physiology, Concordia University, Montreal, QC H4B 1R6, Canada; maryse.fortin@concordia.ca; 15 Herston Imaging Research Facility, University of Queensland, Brisbane, QLD 4072, Australia; g.galloway@uq.edu.au; 16 Department of Physical Therapy, North Central College, Naperville, IL 60540, USA; mahoggarth@noctrl.edu; 17 School of Rehabilitative and Health Sciences, Regis University, Denver, CO 80221, USA; llaporta@regis.edu; 18 Center for Translational Pain Medicine, Department of Anesthesiology, School of Medicine, Duke University, Durham, NC 27710, USA; andrea.nackley@duke.edu; 19 Occupational and Environmental Medicine Centre, Department of Health Medicine and Caring Sciences, Unit of Clinical Medicine, Linköping University, 58183 Linköping, Sweden; anneli.peolsson@liu.se; 20 Department of Health Medicine and Caring Sciences, Unit of Physiotherapy, Linköping University, 58183 Linköping, Sweden; 21 School of Allied Health, La Trobe University, Melbourne, VIC 3086, Australia; z.perraton@latrobe.edu.au (Z.P.); a.semciw@latrobe.edu.au (A.I.S.); 22 Department of Physical Therapy and Human Movement Sciences, Feinberg School of Medicine, Northwestern University, Chicago, IL 60611, USA; amee.seitz@northwestern.edu; 23 School of Medicine, University of Colorado, Aurora, CO 80045, USA; andrew.c2.smith@cuanschutz.edu; 24 Discipline of Physiotherapy, University of Newcastle, Callaghan, NSW 2308, Australia; suzanne.snodgrass@newcastle.edu.au; 25 Adelaide Medical School, University of Adelaide, Adelaide, SA 5005, Australia; viennatran42@gmail.com; 26 School of Health & Social Care, Edinburgh Napier University, Edinburgh, Scotland EH11 4BN, UK; s.valentin@napier.ac.uk; 27 School of Physical Therapy, Western University, London, ON N6A 3K7, Canada; dwalton5@uwo.ca; 28 School of Medicine and Dentistry, Griffith University, Brisbane, QLD 4111, Australia

**Keywords:** artificial intelligence, neural networks, machine learning, MR imaging, muscle fat infiltration, public datasets, normative reference data

## Abstract

Disorders affecting the neurological and musculoskeletal systems represent international health priorities. A significant impediment to progress in trials of new therapies is the absence of responsive, objective, and valid outcome measures sensitive to early disease changes. A key finding in individuals with neuromuscular and musculoskeletal disorders is the compositional changes to muscles, evinced by the expression of fatty infiltrates. Quantification of skeletal muscle composition by MRI has emerged as a sensitive marker for the severity of these disorders; however, little is known about the composition of healthy muscles across the lifespan. Knowledge of what is ‘typical’ age-related muscle composition is essential to accurately identify and evaluate what is ‘atypical’. This innovative project, known as the MuscleMap, will achieve the first important steps towards establishing a world-first, normative reference MRI dataset of skeletal muscle composition with the potential to provide valuable insights into various diseases and disorders, ultimately improving patient care and advancing research in the field.

## 1. Introduction

Many common diseases and disorders, including, but not limited to, cancer, diabetes, Parkinsons, cardiovascular, neuromuscular and musculoskeletal conditions, chronic fatigue syndrome, osteoarthritis, and frailty, lead to progressive muscle weakness that hinders daily activities. The slow progression of these conditions makes it difficult to assess therapeutic interventions because the impact of disease progression often gets masked by age-related changes. In order to develop and implement robust outcome measures capable of accurately assessing the impact of change in muscle composition, the confounding influence of normal age-related changes needs to be comprehensively accounted for. While there are age-related reference values or models of muscle composition (e.g., fat infiltrates) specific to certain muscle groups, they lack generalizability beyond the populations being studied and lack representation across sex and ethnicity [1,2,3,4]. Knowledge of what is ‘typical’ is essential to accurately identify, effectively evaluate, and subsequently treat what is ‘atypical’. Therefore, it is vital to develop reference values for demographic and anthropometric-related muscle composition across the lifespan.

Skeletal muscle composition and morphometry are receiving more attention as advances in machine learning (i.e., supervised deep learning segmentation models) permit improved visualisation and rapid quantification of muscle size and composition in clinically indicated computed tomography (CT) and magnetic resonance imaging (MRI) scans [5,6]. CT and MRI represent routes to potential imaging biomarkers of muscle health. MRI is commonly preferred for quantifying muscle size and composition due to superior soft tissue contrast and lack of ionizing radiation [7,8]. A biomarker that can be measured across the lifespan and benchmarked with normative values would have a profound impact on the field for many neuromuscular and musculoskeletal disorders. They appear to be useful and accurate for measuring the magnitude, distribution, and clinical course of skeletal muscle deterioration [9,10], are independent of patient effort and assessor variability, and can be benchmarked against normative values [11,12]. However, there are no age-related reference values against which to measure relative changes in skeletal muscle over time or in response to therapy. Studies to date are limited to specific muscle groups in age-limited patient populations, are reported using laborious manual data analytics, and lack easily accessible normative reference values to understand the presence, magnitude, and distribution of muscle pathology according to age [11,12,13].

Our primary goal is to establish MuscleMap, an open-source, community-supported consortium for whole-body quantitative MRI of muscle. MuscleMap aims to provide standardised norms for muscle composition and morphometry, incorporating demographic and anthropometric variables through state-of-the-art medical imaging and machine learning technologies. By doing so we aim to provide comprehensive, well-characterised biomarkers of muscle composition that can detect abnormalities early, monitor disease progression, and evaluate therapeutic effectiveness. MuscleMap will enable more rapid, diverse, and equitable advancement in research on many chronic health conditions.

Our commitment to this goal is underscored by (1) what has already been completed, (2) what is currently in progress, (3) what is planned, and (4) the exploration of new directions, spanning diverse body regions and disciplines, encompassing wellbeing and the prediction of optimal health outcomes.

The objectives of MuscleMap are as follows:

(1) Develop a standardized acquisition protocol for whole-body quantitative MRI of muscle for the most common MR manufacturers (General Electric, Siemens, and Philips).
(2) Generate a large (n ≥ 1000) open-source annotated multi-site, multi-racial, and multi-ethnic heterogenous whole-body muscle MRI dataset across the lifespan using MuscleMap’s standardized acquisition protocol.
(3) Create an open-source toolbox for the analysis of whole-body muscle morphometry and composition using the MuscleMap whole-body muscle MRI dataset.
(4) Develop normative models for whole-body human skeletal muscle morphometry and composition with respect to age, sex, gender, site, race, ethnicity, and body habitus using the MuscleMap database.
(5) Identify and quantify changes in skeletal muscle morphometry and composition associated with diseases and disorders, compared to MuscleMap normative models.
(6) Establish the necessary regulatory and data informatics infrastructure for the implementation of the MuscleMap toolbox and normative models into clinical workflows.

## 2. Why Is MuscleMap Needed?

Large muscle imaging datasets are needed to provide insights into what changes in muscle morphology occur with age. MuscleMap, which will be built from a large heterogenous MRI dataset, will provide this information and will deliver it open-source. These normative reference values will be used to (1) diagnose pathology, (2) evaluate the efficacy of interventions, (3) monitor disease severity and progression, and (4) assist in the development of responsive outcome measures for disease-modifying therapeutic trials.

There is a pressing need for an acquisition protocol to ensure high-quality and standardized muscle imaging across the body as the quantification of muscle size and intramuscular fat appears to be dependent on the methodology [14]. Whole-body quantification of muscle composition and size with MRI is challenging due to between-subject variability (e.g., varying size and shape of muscles and people), the need to standardize imaging for each body region (e.g., positioning, field-of-view, image resolution, and shimming), and variability in acquisition protocols (e.g., field strength, imaging parameters, and hardware). For example, differences in imaging techniques (T_2_-weighted versus Dixon fat–water MRI) and positioning of the field-of-view (intramuscular fat varies spatially) affect the measures of muscle health [14,15]. As shown in the spinal cord imaging field [16], a generic acquisition protocol will (a) reduce the resources needed for beginning quantitative muscle MRI and (b) reduce the variability (or increase standardization) in imaging parameters for multi-site and multi-manufacturer studies, improving accuracy and reliability.

Contemporary techniques to quantify muscle composition rely on manual muscle segmentation, which is laborious and precludes large-scale examinations. MuscleMap solves this by curating a large heterogenous open-source dataset of whole-body muscle MRI that will be used to develop generalizable computer-vision models (CVMs) and machine learning algorithms. Similarly, the recent availability of open-source datasets, such as MedicalDecathlon [17], Amos [18], and Chaos [19] is opening avenues for building generalizable machine learning networks that cover wide patient, manufacturer, and demographic (i.e., age, sex, ethnicity etc.) variabilities. One such example of existing large datasets for muscle MRI is the UK Biobank—the world’s largest biomedical research dataset of whole-body MRI and dual-energy X-ray absorptiometry (DEXA) images [20]. However, whilst the UK Biobank is a large prospective cohort study with imaging on over 50,000 males and females, it is limited to ages 40–69 years and includes only people from the United Kingdom who live within 25 miles (40 km) of 22 assessment centres, impacting its generalizability globally [21]. Additionally, the UK Biobank’s imaging protocols were designed to broadly assess muscle and fat across body regions, not within individual muscles, reducing its utility in studying conditions affecting specific muscles. The MuscleMap dataset will provide the global research community with an opportunity to develop a toolbox to accurately, reliably, and rapidly measure muscle composition with sufficient resolution to study muscle-specific ageing and disease processes. Combining the standardized acquisition and analysis methods with a large heterogenous dataset in healthy subjects, MuscleMap will open avenues for normative modelling approaches. This will provide the opportunity to expand the breadth and depth of our knowledge of muscle health across childhood, adolescence, adulthood, senescence, sex, gender, race, and ethnicity so that researchers and clinicians can better interpret and identify deviations that may indicate potential health issues. Normative modelling—quantifying how an individual’s muscle measurements deviate from normative values—will allow for precise and personalized assessments by translating raw data into a meaningful context relative to a reference population. Consequently, normative modelling enhances the ability to detect subtle changes in muscle health, tailor interventions, and track progress over time, ultimately contributing to patient-tailored treatments [22,23]. In the subsequent sections, we provide a non-systematic narrative overview of existing knowledge underpinning the development and completion of the emerging MuscleMap, region by region, starting with the cervical spine and ending with the lower extremity.

## 3. Regional Anatomy and Musculature

### 3.1. Cervical Spine

To provide historical context, MuscleMap started after nearly 10 years of investigation into clinical observations of muscle morphometry (size/shape) and muscle composition (muscle fat infiltration (MFI)) using MRI in patients with chronic pain following whiplash injury from motor vehicle collision [24]. Specifically, we sought to understand the relevance of these observations to the whiplash condition, which might better underpin the assessment and management of these patients.

Using manual methods, MFI can be quantified from conventional (T_1_- and T_2_-weighted) and advanced (Dixon and proton density fat fraction) MRIs and CTs [24,25,26] and has now been consistently observed and reported in patients with idiopathic neck pain, degenerative cervical myelopathy (DCM), and traumatic spinal cord injury [27,28,29]. Despite the multifactorial aetiology of these conditions, there is a characteristic pattern of MFI whereby deep muscular layers of the cervical extensors and cervical flexors [25,30] have the greatest magnitude of MFI and reductions in cross-sectional area in people with chronic post-traumatic neck pain. MFI is also a significant predictor of poor functional recovery and either directly involved in, or associated with, the biological mechanisms underlying persistent neck-related disability, making it a potential target for treatment. Therefore, assessing the magnitude of MFI could alter management and potentially improve rates of recovery from persistent spinal disorders [31].

Manual segmentation of spinal muscles is not routinely performed clinically, thus limiting its use in research environments. Accordingly, we originally trained a deep learning CVM to perform segmentation of a single muscle group (multifidus and semispinalis cervicis) [32], and later multiple muscles [5], in participants with varying levels of neck pain and disability. Research in the area also demonstrates that MRI-based changes in muscle morphology and composition can inform management. In one study, people with idiopathic neck pain were observed to have a significantly smaller cross-sectional area (CSA), suggesting atrophy, in 8 of 14 muscle regions examined compared to controls, signalling potentially important targets for interventions to target muscle hypertrophy in the management of neck disorders [33,34].

**Figure 1:**
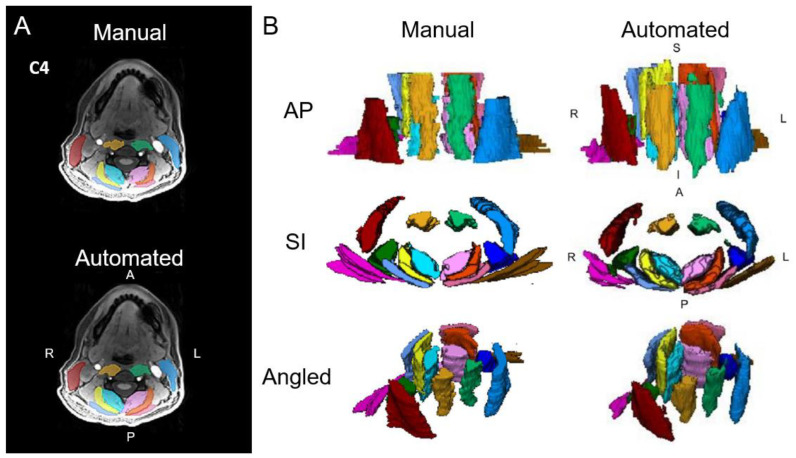
(**A**) Axial cervical spine muscle segmentations at the C4 vertebral level from manual segmentation and an automated computer-vision model overlaid over a water image from Dixon fat–water MRI. (**B**) Three-dimensional renderings of cervical spine muscle segmentations. The muscle groups segmented include the multifidus and semispinalis cervicis (left = light pink, right = aqua), longus colli and longus capitis (left = light green, right = gold), semispinalis capitis (left = orange, right = yellow), splenius capitis (left = dark pink, right = light blue), levator scapula (left = indigo, right = dark green), sternocleidomastoid (left = blue, right = red), and trapezius (left = brown, right = magenta). L = left, R = right, A = anterior, P = posterior, S = superior, I = inferior. Adapted from Weber et al., 2021 [5].

Our success in automatically segmenting the muscles traversing the cervical spine, with its architecturally complex anatomy, indicates that effectively extending these methods to other body regions is possible and warranted. Building on the success of our studies on the cervical spine, we have also received several imaging datasets from other consortium members to complete the buildout of automatic segmentation algorithms for the muscles traversing the lumbar spine, and efforts are underway for muscles involved in deglutition, as well as foot, leg, hip, shoulder, and pelvic floor muscles using both CT and MRI. The automatic segmentation of skeletal muscles using CVMs solves the long-standing problem of time-dependent manual techniques, permitting rapid and accurate quantitative comprehensive assessment of the cervical spine [5] [see Figure 1A,B] and other muscles throughout the human body at near-human accuracy.

### 3.2. Muscles Involved in Deglutition

Previous MRI research has investigated the biomechanics of normal and age-related swallowing [35] and quantified changes in muscles in the pharynx, larynx, and oral cavity in clinical populations such as head and neck cancers [36,37] and whiplash-associated disorders [38]. To date, research has been largely retrospective, single-site studies involving manual contouring of swallowing muscles/structures. This is time-intensive and dependent on requisite skills and the rigour of local quality assurance processes [39], limiting their generalizability and clinical utility. Early work demonstrated the feasibility of developing deep learning-based models for auto-segmentation of swallowing-related structures based on CT images [40]; however, this process has yet to be applied to clinically available MRIs. Similar to MFI, markers of impaired swallowing may aid clinical care, identifying specific patients who would benefit from further assessment and/or rehabilitation to optimise oral intake and preserve nutrition.

### 3.3. Shoulder

Shoulder pain is the third most common form of musculoskeletal disorder, with the two most common conditions being rotator cuff (RC) tears and glenohumeral osteoarthritis. The prevalences of both RC and glenohumeral osteoarthritis are increasing, with symptomatic RC tears in ~20% of the population [41] and the lifetime risk of glenohumeral osteoarthritis surgery doubling since 2007 [42]. Coupled with a concerning six-fold increase in RC surgery rates [43], the annual cost of RC-related surgeries in an ageing population across Australia has increased by more than 200% over the past two decades [44], with similar figures reported in the United States [45] and the UK [46]. An ageing population suggests this problem will only continue to rise.

Skeletal muscle composition is a strong predictor of failure rates following RC repair [47]. MFI also serves as a critically important biomarker in the functional recovery of people living with glenohumeral osteoarthritis after surgery [48,49]. However, describing associations between muscle composition and patient outcomes is complex because normative data for shoulder muscle composition across the lifespan has not been well characterized and often confounds comparative and longitudinal analysis of disease progression [50]. Variations in demographic characteristics (age, sex, ethnicity, and co-morbidities) [51] also hinder the interpretation and application of this research. Deep learning techniques to automate the quantification of shoulder MFI will facilitate the development of machine learning models while training to predict patient outcome and recovery and relieve the RC and glenohumeral osteoarthritis burden of disease in our healthcare systems. Work is currently underway to establish a shoulder CVM for muscles of the rotator cuff.

### 3.4. Lumbar Spine

**Figure 2:**
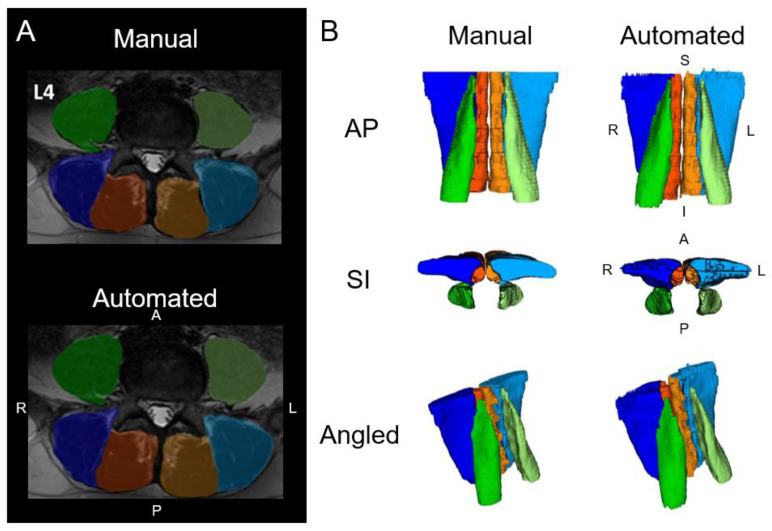
(**A**) Axial lumbar spine muscle segmentations at the L4 vertebral level from manual segmentation and an automated computer-vision model overlaid over a spin-echo T_2_-weighted image. (**B**) Three-dimensional renderings of the lumbar spine muscle segmentations. The muscle groups segmented include the multifidus (left = light orange, right = dark orange), erector spinae (left = light blue, right = dark blue), and psoas major (left = light green, right = dark green). L = left, R = right, A = anterior, P = posterior, S = superior, I = inferior. Adapted from Wesselink et al., 2022 [6].

Low back pain (LBP) is the leading cause of years lived with disability and its vast financial cost and global impact on population health drives the study of the lumbar spine [52]. Activity-limiting LBP affected 619 million people globally in 2020 and is projected to increase by over a third in 2050, largely driven by ageing and detrimental lifestyle factors (i.e., lack of physical activity, smoking, and larger body habitus) [53]. Around 90% of LBP is non-specific, where salient structural pathology that relates to clinical symptoms is elusive [54]. Improved imaging and machine learning analysis methods for lumbar paraspinal muscles [55,56] reveal promising distinctions between people with spinal disorders [6,57] (Figure 2).

Typical morphological ageing of the lumbar spine muscles has not been well characterized [58], with a paucity of longitudinal data [59,60] or reports factoring established confounders like age, sex, BMI, and ethnicity [12,61,62]. The spatial distribution of lumbar MFI appears influential, with higher fat content in the lower lumbar levels [61] in the deeper multifidus fibres abutting bone (versus erector spinae or psoas) [60] and in relation to the lumbar centre of motion [63].

Attracting mounting interest is the clinical importance of lumbar paravertebral muscle morphology and whether a decline in muscle composition is reversible with physical activity. Important research examining the influence of spaceflight, micro-gravity, or bedrest on muscle morphology is contributing to the mechanistic understanding of lumbar muscle function. For example, multifidus responded inhomogeneously to 60 days of bed rest in healthy volunteers and may indicate a susceptibility specific to the shortest multifidus fibres [60]. Observing and reliably measuring the temporal change in lumbar paraspinal muscles is a complex and challenging endeavour. While in its relative infancy, efforts are being excitingly enabled by ever-evolving imaging and analysis technologies and global collaboration like MuscleMap.

### 3.5. Pelvic Floor

Conventional imaging techniques have limited ability to distinguish between the contractile and non-contractile components of individual muscles in the pelvic floor. This information is essential to improve our understanding of how normal muscle composition is impacted in patients with pelvic floor dysfunction. Environmental factors such as age and injury (e.g., birth trauma to the pelvic floor muscles), and genetic factors such as polymorphisms in collagen (e.g., COL3A1) and matrix metalloproteinase (e.g., MMP1) genes [64] that lead to a loss of pelvic floor muscle function are associated with increased risk of disorders, including urinary incontinence, faecal incontinence, sexual dysfunction, and pelvic organ prolapse. The development of a CVM for the pelvic floor will provide a reference for clinicians, with an improved understanding of age-related changes and ethnographic factors so as to ultimately improve clinical reasoning and choice of therapeutic interventions. The ability to evaluate pelvic floor architecture rapidly and accurately in looking at physiological age-related changes as well as determining the success of therapeutic interventions will be important.

### 3.6. Gluteal Muscles

The relationship between hip muscle size and osteoarthritis of the hip has been shown to be related to activity and clinical severity of the clinical course [65,66]. As in other areas of the body, challenges towards untangling the causes of, or contributors to, muscular changes observed in those with hip pathology, remain. Results of a recent 60-day bedrest study involving healthy participants may shed some light on this issue [67] as reductions in muscle volumes of the gluteus maximus (~10%), gluteus medius (8%), and gluteus minimus (10.5%) were observed.

**Figure 3:**
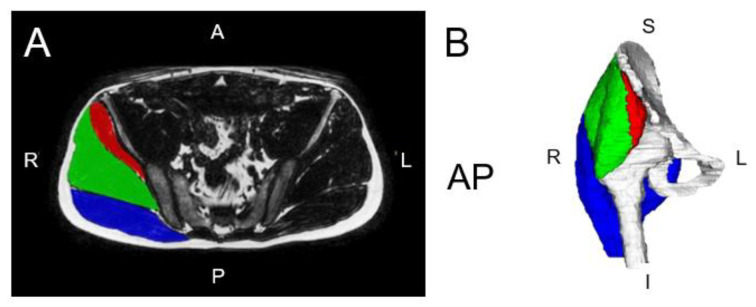
(**A**) Axial right hip muscle segmentation overlaid over a fat image from Dixon fat–water MRI. (**B**) Three-dimensional renderings of the hip muscle segmentations. The muscle groups segmented include the gluteus maximus (blue), gluteus medius (green), and gluteus minimus (red). Adapted from Perraton et al., 2024 [69].

Members of our team recently performed a scoping review of hip muscle segmentation and identified that research in the field is growing exponentially [68]. We also identified up to seven different anatomical landmarks that have been reported on and used for measurement of the cross-sectional area or MFI of the hip muscles. We encourage authors of future work to be consistent with these landmarks to better build consistent automatic segmentation models for clinical use. Figure 3A,B detail the developing CVM for the gluteal muscles.

### 3.7. Thigh and Leg Musculature

Muscle volume and MFI of the thigh and leg musculature continue to be investigated for their potential association with health outcomes such as insulin resistance, metabolic abnormalities, cardiovascular disease, knee osteoarthritis, frailty, and mortality [70,71,72]. Anti-gravity muscles of the lower extremity, including the quadriceps femoris and gastrocnemius, regulate posture and gait. Their function is often impaired by ageing and neuromuscular disorders such as Duchenne muscular dystrophy (DMD), Charcot–Marie–Tooth disease (CMT) [73,74], and spinal muscular atrophy (SMA) [75]. Quantitative MRI studies show significant infiltration of the thigh and leg in CMT1A patients and a combination of atrophy and infiltration of the thigh in SMA 2 and 3 patients. Such studies primarily rely on manual segmentation methods and small cohorts, which, as stated, are not time-efficient and are limited to the research landscape.

### 3.8. Foot and Ankle

Foot and ankle muscle dysfunction has been linked to a variety of common clinical conditions. Imaging methods have been used to demonstrate foot muscle morphology changes in people with progressive neuromuscular conditions such as CMT [76], diabetic neuropathy [77], plantar heel pain [78], ankle instability, and hallux valgus [79]. The relationship between foot muscle morphology and chronic conditions supports a need to identify changes in foot muscle structure and composition over time.

**Figure 4:**
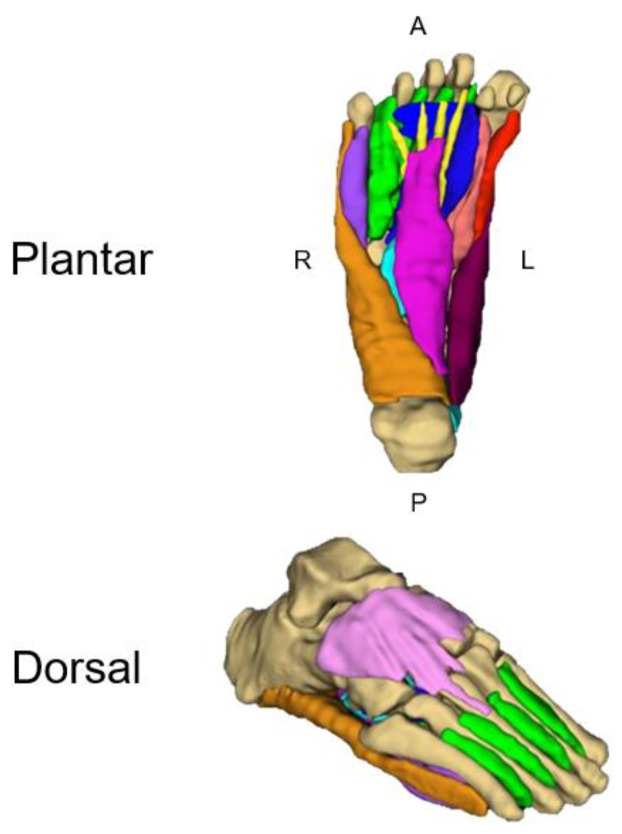
Three-dimensional renderings of the intrinsic foot muscles from Dixon fat–water imaging. The muscle groups segmented included the abductor hallucis (plum), quadratus plantae (light blue), flexor digitorum brevis (fuchsia), abductor digiti minimi (orange), lumbricals (yellow), extensor digitorum brevis (pink), flexor hallucis brevis medial head (red), flexor hallucis brevis lateral head (salmon), adductor hallucis (dark blue), flexor digiti minimi (purple), and plantar and dorsal interossei (green). Adapted from Franettovich et al., 2021 [80].

However, the 6–7 h required to manually segment the individual intrinsic foot muscles on MRI has limited any capacity to leverage advancing acquisition techniques at higher field strengths and implement measurement into clinical practice (Figure 4). Progress towards overcoming these time-consuming demands has been made by manually measuring every tenth slice, reducing the segmentation time to ~30 min without sacrificing accuracy [80]. While this method provided substantial time savings, it does not overcome the demands for large samples, time challenges for a busy radiology practice, or the assessment of multiple muscle groups. Accordingly, we are working to expand our MuscleMap efforts to develop a deep learning CVM to automate the segmentation of the intrinsic foot muscles. Preliminary results show a reduction in the time required for segmentation of the intrinsic foot muscles to less than 30 s, representing a remarkable improvement in efficiency, feasibility, and implementation.

## 4. Conditions and Disorders

### 4.1. Spinal Cord Injury

Imaging has long been used to quantify skeletal muscle morphology in people with spinal cord injury [81]. As an expected consequence of reduced volitional muscle activation caudal to the level of injury, MRI and CT markers of muscle atrophy such as MFI have been reported [28,82,83]. While atrophy and MFI progress after the initial injury [82], community ambulators tend to have larger muscles with less MFI compared to users of a wheelchair [83,84]. In the spinal cord injury population, interventions such as resistance training, locomotor training, and neuromuscular electrical stimulation attenuate lower extremity skeletal muscle atrophy [85]. Semi-automated segmentation using a threshold approach as well as more automated CVM approaches have been explored in this population, reducing the time required to quantify and monitor the composition, size, and shape of skeletal muscle [86,87]. MuscleMap expands this work, providing an automated tool for quantitative measurement of impairment, monitoring progression over time and evaluating the effects of new treatments in clinical trials of those with spinal cord injury.

### 4.2. Sarcopenia and Frailty

Progressive loss of muscle mass accompanied by a decline in muscle function are cardinal features of sarcopenia; however, key features remain unclear and our understanding of precisely how this disease progresses is incomplete. Contemporary investigations into the biological mechanisms responsible for sarcopenia have identified drivers such as decreases in motor neuron numbers possibly driven by loss of nuclear envelope integrity [88]. However, how these alterations relate to normal lifespan alterations in muscle volume and function remains largely unclear. Studies of muscle fibre function, volume, and spatial distribution [89] highlight the technical difficulties in generating data that allow meaningful analysis of age-related changes in skeletal muscle in small samples or individual muscle groups.

There is a high prevalence of sarcopenia in frailty, which contributes to a state of vulnerability and loss of systemic homeostasis to external stressors [90]. The loss of homeostasis is related to reduced physiologic reserve in a range of systems (e.g., neurological, endocrine, immune, muscle, cardiovascular, respiratory, renal, and bone), which increases with age [90]. This is related to ageing physiology, frailty physiology, reduced physical activity, increased prevalence of multi-morbidity, and high medication use with side effects that directly impair muscle and those that indirectly impair muscle by reducing physical activity or nutrition.

Current best practices for the management of frailty include identification of frailty, prescription of exercise, dietary interventions, and medication review. The emerging field of geroscience [91] is developing interventions that target the ageing process itself and could prevent or reduce age-related chronic diseases and frailty. Quantitative MRI measurements in small samples correlate frailty and muscle function across different age groups [72] and MuscleMap will permit the detection of responses to interventions targeting ageing and frailty.

#### Degenerative Cervical Myelopathy

Recent studies conducted by members of our team have investigated the cervical musculature in patients with DCM and reported associations between poor muscle quality, clinical outcomes, and muscle function [27,31,92]. Improving our current knowledge regarding the characteristics and implications of cervical muscle morphology in DCM and other cervical disorders may provide useful insights for more effective and informed rehabilitation approaches. The tedious and rater-dependent nature of manual segmentation methods for the assessment of cervical muscle morphology and composition provides the impetus for MuscleMap, which will allow for open-access automated segmentation of cervical muscles from commonly used CT and MRI sequences.

### 4.3. Osteoarthritis

In 2019, the global burden of disease reported osteoarthritis as the most common form of arthritis, affecting about 6% of the global population or more than 500 million people worldwide. It is ranked as the 15th highest cause of years lived with disability, and most commonly affects the knee [93]. The muscles of the thigh are crucial to the biomechanical stability and load distribution of the knee, so it follows that the structural changes and/or weaknesses of these muscles have long been associated with the development [94] and progression of osteoarthritis [95,96]. Despite the important role that muscle structure and function play in osteoarthritis, little is known about the changes in muscle composition that occur over the disease course; however, decreases in quadriceps CSA and increases in MFI have been shown to be associated with downstream worsening of knee osteoarthritis symptoms and greater risk of future knee replacement surgery [97]. This area of research is still in its infancy and MuscleMap will help to address important knowledge gaps pertaining to the structure of muscles across the disease course compared with age-related normative data and may identify new avenues for muscle-directed disease-modifying interventions for osteoarthritis of the knee and other joints such as the hip, hand, shoulder, foot, ankle, and spine.

### 4.4. Diabetes

Over half a billion people live with diabetes worldwide [98]. Both type 1 and type 2 diabetes are associated with relatively lower lean muscle mass to total body weight ratios compared to the general population [99]. Hyperglycaemia, peripheral insulin resistance, dyslipidaemia, hypertension, and obesity are common to both forms of diabetes, and these factors have detrimental effects on the skeletal muscle, causing changes in muscle fibre composition, metabolism, insulin sensitivity, ATPase activity, and mitochondrial function. Furthermore, type 2 diabetes can directly result from skeletal muscle abnormalities, indicating a bidirectional relationship [100].

Diabetes can directly affect muscle integrity and strength, as evidenced by diabetic amyotrophy, myopathy, and peripheral neuropathy. Furthermore, conditions such as frozen shoulder, osteoarthritis, diffuse idiopathic skeletal hyperostosis, and Duputryen’s contractures are more common in people with diabetes [101]. Long-term muscular, joint and nerve-related syndromes can lead to chronic pain and disuse, further exacerbating muscle wasting and contributing to sarcopenia. While these conditions are well characterised by advanced imaging techniques, there remain a number of questions that MuscleMap will aim to answer. These include, but are not limited to, the identification of features on MRI that can predict the development of diabetes-related muscle disorders or the chronicity of pain-related syndromes and whether anti-diabetic glucose-lowering therapy is superior to glucagon-like peptide 1 receptor agonists at protecting/restoring muscle mass.

### 4.5. Cancer

Cachexia is not unique to, but is a common syndrome associated with, more than half of patients diagnosed with cancer and up to 80% of patients with advanced disease [102]. The hallmarks of cachexia include systemic inflammation, altered metabolism, reduced nutrition, and muscle wasting [93] and are particularly prevalent in pancreatic and gastrointestinal cancers. The introduction of surgery and chemotherapy to patients who are already cachexic amplifies the problem and is associated with worse survival outcomes [103].

Although it can be difficult to predict which patients will suffer from cachexia, diagnosis and early intervention are critical due to its known association with poor prognosis [101]. Whilst measurements of albumin and inflammatory markers in cancer patients are useful as indicators of the degree of nutrition and inflammation, respectively, they do not provide quantitative data on muscle volume or compositional changes. MuscleMap will be critical for identifying baseline muscle composition and changes that occur during treatment for cancer. Through the incorporation of these muscle markers with routine biological markers, we have the potential to not only provide important prognostic information but also to identify patients in whom early intervention may result in clinically meaningful survival outcomes.

### 4.6. Incontinence

Pelvic floor muscle and connective tissue decline play a role in the development of urinary, faecal, and double incontinence as well as sexual dysfunction and pelvic organ prolapse. The prevalence of urinary incontinence is high and increases with age. A total of 2.3 million global citizens verbally report lower urinary tract symptoms, with nearly 50% being males [104]. Conservative treatment of urinary incontinence focuses on strengthening the pelvic floor muscles to support the bladder and bowel (and uterus). There is strong evidence that exercise can positively influence symptoms and improve the quality of life in all types of urinary incontinence. However, if muscle decline is irreversible, other treatment strategies, including surgery, may be indicated. Hence, the status of muscle composition with respect to ageing and sex, in comparison to normative values, would help clinicians make informed decisions on appropriate therapeutic interventions.

Faecal incontinence is a distressing condition that affects nearly 10% of adults in community settings [105]. The prevalence of faecal incontinence increases with age and affects women and men equally [106]. Pelvic floor injury is a well-recognised risk factor for faecal incontinence, with the most common cause being obstetric trauma damage to the anal sphincters, particularly associated with instrumental vaginal delivery. Anal surgery, which involves sphincter division, and perineal or pelvic trauma can also result in faecal incontinence. Pelvic organ prolapse is a common condition in which the pelvic organs prolapse into the vagina, impacting sexual, urinary, and defecatory functions and quality of life [107]. Whilst MRI is used clinically to identify structural issues in pelvic organ prolapse, the assessment of pelvic floor muscle parameters in patients with faecal incontinence or urinary incontinence has been underexplored [108].

## 5. Conclusions

MuscleMap will overcome the current difficulty of detecting atypical changes in muscle health and provide comprehensive, well-characterized biomarkers of muscle composition and size across the lifespan. This objective, unbiased measure may (1) facilitate elucidating changes earlier than commonly used clinical outcome measures, (2) be correlated with clinical endpoints, (3) provide a clinically applicable screening tool for at-risk populations, and (4) control for sociodemographic modulators. This is likely to result in substantial breakthroughs in the understanding and availability of promising new therapies for devastating and debilitating diseases and disorders that affect muscle health.

Similar to the Human Genome Project [109], MuscleMap is another example of applied systems design in biology, leading to new multidisciplinary collaborative ways of examining the linkages and interactions between disparate multimodal datasets. This is only possible through a project of this scale whereby the extraction of large amounts of representative image-derived features of muscle has the potential to uncover disease or disorder characteristics that fail to be captured with current assessment techniques.

The integration of automatic segmentation post-processing computer-vision models into both the conventional standard-of-care clinical workflow and images already in a patient’s medical record is relatively straightforward, and in the not-too-distant future, these methods should provide clinicians, patients, and researchers with quantitative metrics of muscle health. These muscle-related measures would complement examination and standard imaging findings and may provide increased diagnostic, prognostic, and predictive information to better inform the assessment and management of individuals with a wide variety of diseases/disorders.

MuscleMap adopts a data-driven open-sourced methodology to iteratively enhance the efficacy of computer-vision models tailored for the analysis of MRI across MR manufacturers. In particular, a multi-disciplinary/institutional collaboration for processing annotated image datasets will badge the future generalizability and efficiency of computer-vision models for unseen datasets. The standardized MuscleMap acquisition protocol and toolbox, aimed at simplifying the use of muscle segmentation software, will be made readily accessible to facilitate the application of these models, freely accessed here https://github.com/MuscleMap/MuscleMap URL (accessed on 17 October 2024) with details on how the MRI community can contribute. It is anticipated that the process will evolve and updates will occur on a frequent and well-communicated basis.
